# Evofosfamide Is Effective against Pediatric Aggressive Glioma Cell Lines in Hypoxic Conditions and Potentiates the Effect of Cytotoxic Chemotherapy and Ionizing Radiations

**DOI:** 10.3390/cancers13081804

**Published:** 2021-04-09

**Authors:** Quentin Bailleul, Pauline Navarin, Mélanie Arcicasa, Christine Bal-Mahieu, Angel Montero Carcaboso, Xuefen Le Bourhis, Alessandro Furlan, Samuel Meignan, Pierre Leblond

**Affiliations:** 1 Unité Tumorigenèse et Résistance aux Traitements, Centre Oscar Lambret, 59000 Lille, France; q-bailleul@o-lambret.fr (Q.B.); pauline.navarin@chru-lille.fr (P.N.); m-arcicasa@o-lambret.fr (M.A.); C-bal@o-lambret.fr (C.B.-M.); a-furlan@o-lambret.fr (A.F.); 2 UMR9020-U1277—CANTHER—Cancer Heterogeneity Plasticity and Resistance to Therapies, CHU Lille, University Lille, CNRS, Inserm, 59000 Lille, France; xuefen.le-bourhis@univ-lille.fr; 3 Service d’Onco-Hématologie Pédiatrique, CHU Lille, 59000 Lille, France; 4 Institut de Recerca Sant Joan de Deu, 08950 Barcelona, Spain; amontero@fsjd.org; 5 Département de Cancérologie Pédiatrique, Institut D’hématologie et D’oncologie Pédiatrique, 69008 Lyon, France; pierre.leblond@ihope.fr

**Keywords:** high-grade glioma, Evofosfamide, hypoxia, radiotherapy, child, DIPG

## Abstract

**Simple Summary:**

Despite many therapeutic approaches attempted over the last years, the prognosis of children with high-grade glioma or diffuse intrinsic pontine glioma remains dismal. Hypoxia-activated prodrugs (HAPs) were developed to target hypoxic areas within solid tumors as gliomas. Evofosfamide (Evo) is a 2nd generation HAP exhibiting significant preclinical and clinical activities against adult glioblastoma. We thus investigated the potential of Evo in six pediatric glioma cell lines. Interestingly, we showed that the growth of all cell lines was inhibited by Evo, mainly under hypoxia as expected. We also evidenced a significant synergism between Evo and three drugs widely used in pediatric oncology. Finally, Evo appeared able to potentiate the effect of ionizing radiations. Since these tumors are highly hypoxic and Evo appears effective in hypoxic glioma cells as a single drug and in combination with radio- and chemotherapy, hypoxia-activated prodrugs could represent a promising therapeutic option for children with brain tumors.

**Abstract:**

Hypoxia is a hallmark of many solid tumors and is associated with resistance to anticancer treatments. Hypoxia-activated prodrugs (HAPs) were developed to target the hypoxic regions of these tumors. Among 2nd generation HAPs, Evofosfamide (Evo, also known as TH-302) exhibits preclinical and clinical activities against adult glioblastoma. In this study, we evaluated its potential in the field of pediatric neuro-oncology. We assessed the efficacy of Evo in vitro as a single drug, or in combination with SN38, doxorubicin, and etoposide, against three pediatric high-grade glioma (pHGG) and three diffuse intrinsic pontine glioma (DIPG) cell lines under hypoxic conditions. We also investigated radio-sensitizing effects using clonogenic assays. Evo inhibited the growth of all cell lines, mainly under hypoxia. We also highlighted a significant synergism between Evo and doxorubicin, SN38, or etoposide. Finally, Evo radio-sensitized the pHGG cell line tested, both with fractionated and single-dose irradiation schedules. Altogether, we report here the first preclinical proof of evidence about Evofosfamide efficiency against hypoxic pHGG and DIPG cells. Since such tumors are highly hypoxic, and Evo potentiates the effect of ionizing radiation and chemotherapy, it appears as a promising therapeutic strategy for children with brain tumors.

## 1. Introduction

Brain tumors are the most frequent solid tumors in children and adolescents [1]. Among them, high-grade glioma (HGG) and diffuse intrinsic pontine glioma (DIPG) certainly count among the most significant challenges of pediatric oncology. Pediatric HGG (pHGG), and principally DIPG, are well known for demonstrating very different biological profiles compared to adult HGG, making the extrapolation of data obtained from adult patients inappropriate and requiring the achievement of specific pediatric studies [2,3,4]. Therefore, pHGG and DIPG remain a formidable challenge for pediatric oncologists. Indeed, despite numerous clinical trials, the prognosis of children with pHGG or DIPG remains very poor, and the median survival for patients with DIPG is currently no longer than 12 months. DIPGs appear unresectable due to their location to the pons and to their diffuse spread into the near brain tissue. They are highly resistant to conventional cytotoxic agents and poorly sensitive to radiation therapy, which remains the standard of care. Patients with pHGG undergo surgical resection when feasible, followed by radiation therapy and often by alkylating chemotherapy such as temozolomide [5]. However, with less than 20% of a response to chemotherapy, the pHGG chemosensitivity remains very low [6].

A major cause of this resistance to treatments could be the presence of hypoxic areas in the tumor, which is a hallmark of HGG. Indeed, hypoxia is well known to play a central role in tumor aggressiveness through resistance to radiotherapy and chemotherapy. Thereby, hypoxia has appeared as a negative prognostic factor in several clinical studies [7,8,9]. While physiological oxygen concentrations in a healthy brain vary from 0.5% to 7.5%, most of the HGGs are characterized by concentrations lower than 2.5% O_2_, with the presence of extreme severe hypoxic areas around 0.1% O_2_ [10]. 

In this context, strategies have been developed to target hypoxic tumor cells specifically. Among these, hypoxia-activated prodrugs (HAPs), weakly cytotoxic in oxygenated tissues, are designed to generate a more cytotoxic compound, specifically in a low O_2_ environment [11]. Several HAPs are currently in clinical trials for patients with different cancers, including adult HGG. Targeting hypoxic cells is thereby considered as a potentially promising strategy in the specific field of pediatric brain tumors [12].

Among second-generation HAPs, Evofosfamide, also called Evo or TH-302 (Threshold Pharmaceuticals), is maximally activated under deep hypoxic conditions. Evo is a 2-nitroimidazole prodrug generating a cytotoxic bromo-Isophosphoramide mustard (Br-IMP) as follows: Evo is reduced at the nitroimidazole site by ubiquitous cellular reductases such as the flavoprotein cytochrome P450 oxidoreductase (POR). This reduced state persists in the absence of oxygen and leads to a fragmentation releasing the alkylating agent Br-IMP [13]. Evo may also exert a local cytotoxic bystander effect by diffusion to the adjacent less-hypoxic tumor cells [14]. 

Preclinical studies have evaluated the therapeutic effects of Evo used as a single agent or in combination with conventional cytotoxic drugs in different cancers [15,16]. Notably, it has been shown that Evo displays an anticancer activity in a hypoxia-dependent manner and can improve the efficiency of some cytotoxic drugs when used in combination.

Several phase I and II clinical trials have demonstrated the safety and preliminary efficacy of Evo when used as a single agent as well as in combination with multiple conventional agents such as doxorubicin in adult patients [17,18,19]. Interestingly, the progression-free survival (PFS), tumor response, and CA 19-9 (Carbohydrate Antigen 19-9) response were significantly improved in patients with locally advanced pancreatic cancer when treated with Evo in combination with gemcitabine compared with gemcitabine alone [20]. In adults with refractory glioblastoma multiform, a phase I trial associating Evo and bevacizumab showed a 17.4% objective response. Results from the phase II trial are pending.

Here, we evaluate for the first time the Evofosfamide cytotoxic potential against malignant pediatric glioma cell lines when used as a single agent or in combination with ionizing radiations or common cytotoxic drugs that are widely used in pediatric oncology.

## 2. Materials and Methods

### 2.1. Cell Culture and Molecules

Human pediatric high-grade glioma cell lines, SF188, KNS42, and UW479, were kindly provided by Chris Jones (The Institute of Cancer Research, Sutton, UK). All pHGG cell lines were grown as a monolayer in DMEM (Dulbecco’s Modified Eagle Medium) medium supplemented with 10% fetal bovine serum (FBS), L-glutamine, non-essential amino acid solution, and an antibiotics cocktail (Life Technologies, Grand Island, NE, USA). DIPG cell lines, HSJD-DIPG007, HSJD-DIPG013, and HSJD-DIPG014 were kindly supplied by Angel Montero Carcaboso (Hospital Sant Joan de Déu, Barcelona, Spain). DIPG cells were maintained as tumorospheres in the tumor stem medium (TSM), composed half of the Neurobasal™-A medium (Life Technologies) and half of the DMEM/F12 medium, supplemented with 1% of GlutaMAX, 1% antibiotic antimycotic, 1% MEM (Minimum Essential Media) non-essential amino acids, 1% 100 mM sodium pyruvate, and 1% HEPES (4-(2-hydroxyethyl)-1-piperazineethanesulfonic acid) buffer. This medium was extemporaneously supplemented with 2% B-27 supplement (Life Technologies), 20 ng/mL human EGF (Epidermal Growth Factor), 20 ng/mL human basic-FGF (Fibroblast growth factor), 10 ng/mL human PDGF-AA (Platelet-derived growth factor-AA), 10 ng/mL PDGF-BB (Platelet-derived growth factor-BB) (all from Peprotech, Rocky Hill, NJ, USA), and heparin. All cell lines were free of mycoplasma contamination. Cells were incubated in a humid atmosphere at 37 °C with 5% CO_2_. 

Oxygenation parameters were modified using a hypoxia station (Don Witley Hypoxystation H35). Cells were incubated for 24 h under 1% O_2_ before experiments were conducted under hypoxic conditions.

Evofosfamide was kindly provided by Threshold Pharmaceuticals. Doxorubicin, etoposide, and SN38 were purchased from Sigma-Aldrich (St. Louis, MO, USA).

### 2.2. Western Blot Analysis

For the Western blot analysis of hypoxia inducible factor-1α (HIF-1α), cells were incubated in 21% or 1% O2 for 72 h. Briefly, samples were collected in RIPA (RadioImmunoPrecipitation Assay) lysis buffer (50 mM Tris Hydrocloride, pH 7.9, 120 mM NaCl, 1% Nonidet, 1 mM EDTA, 5 mM NaF, 1 mM sodium othovanadate, 0.04 mM 4-(2-Aminoethyl)benzenesulfonyl fluoride hydrochloride), and protein concentration was determined by BCA Assay (BicinChoninic Acid)(Bio-Rad, München, Germany). Western blot analysis was performed using the following antibodies: mouse anti-HIF-1α diluted ^1^/_500_ (BD Bioscience, San Jose, CA, USA) and rabbit anti-actin diluted ^1^/_5000_ (Santa Cruz, Dallas, TX, USA).

### 2.3. Growth Inhibition Assay

Cells were seeded in 96-well plates (5 × 10^3^ cells/well for KNS42, 4 × 10^3^ cells/well for UW479, 1.5 × 10^3^ cells/well for SF188, and 1 × 10^4^ cells/well for DIPG cells) and placed under hypoxic (1% O_2_) or normoxic (21% O_2_) conditions. After 24 h, cells were treated with increasing concentrations of compounds (from 50 nm to 100 µM). After 72 h of incubation under 1% or 21% O_2_, the proportion of viable cells was revealed by the addition of Resazurin for 4 h at 37 °C in the dark. Absorbance was read at 570 and 595 nm using a spectrophotometer (SPECTRAmax, Molecular Devices), and the average absorbance differential (OD 570 nm value–OD 595 nm value) was established for each condition. IC50 values were obtained from the cytotoxicity curves given the percentage of viable cells as a function of the compound concentration. IC50 in hypoxia has been calculated based on the untreated hypoxic condition.

### 2.4. Apoptosis/Necrosis Detection

SF188 and HSJD-DIPG007 cells were incubated for 48 h with or without 20 µM Evo under 1% O_2_ hypoxia as monolayer in a LabTek chamber slide. Then, cell death analysis was assessed using an apoptosis/necrosis detection kit according to manufacturer instructions (Enzo Life Sciences, Lausen, Switzerland). Briefly, cells were washed then incubated with a buffer containing Annexin V-cyanine 3 and the necrosis detection reagent (NDR), a far-red emitting DNA-intercalating dye. Cells were observed with a confocal microscope (LSM880, Zeiss). Cells undergoing apoptosis appeared Annexin V-cyanine 3 positive and NDR negative, while cells undergoing late-stage apoptosis and early necrosis were Annexin V-cyanine 3 and NDR positive. For cell cytometry analysis, cells were treated with IC50 Evo concentration for 72 h under 1% O_2_ hypoxia. Then all cells were collected, labeled using the apoptosis/necrosis detection kit, and analyzed by flow cytometry using a 488 nm laser with fluorescence detection with FL2 (apoptosis detection reagent) and FL3 (NDR) channels (Cytoflex, Beckman Coulter). Cells undergoing apoptosis appeared Annexin V-cyanine 3 positive and NDR negative, while cells undergoing late-stage apoptosis and early necrosis were Annexin V-cyanine 3 and NDR positive.

### 2.5. Drug Synergy Evaluation

Growth inhibition induced by drug combinations was assessed in vitro using Resazurin assay as previously described. Experiments were performed with Evo combined with etoposide, doxorubicin, or SN38 (active metabolite of irinotecan). IC_50_ values of etoposide, doxorubicin, and SN38 were determined beforehand (Figure S3). The cells were seeded in 96-well microtiter plates as previously described and placed under hypoxia (1% O_2_). After 24 h, cells were treated with increasing concentrations of compounds (1/16- to 8-fold IC_50_), alone and in combination at equitoxicity concentration. After the cell viability measurement for each condition, the combination index (CI) was calculated according to Chou and Talalay’s method using CalcuSyn software (Biosoft) at the three dose effects (ED) ED50, ED75, and ED90 (which are dose effect 50, 75, and 90 of a drug combination) [21]. The CI value is the quantitative measure of the degree of drug interaction in terms of antagonistic effect (CI > 1), additive effect (CI = 1), moderate synergism (0.7 < CI < 1), synergism (0.3 < CI < 0.8), or strong synergism (CI < 0.3). Experimental measurements were considered as significant if r > 0.9.

### 2.6. Clonogenic Assay

SF188 cells were seeded into 12-well culture plates (225 cells/well) and placed under hypoxia (1% O_2_). After 24 h of incubation at 37 °C, cells were treated with 0, 0.05, or 0.1 µM Evo. Six hours later, cells were irradiated with fractions of 0, 1, or 2 Grays per day for three consecutive days or with 0, 2, 4, 6, or 8 Grays as a single fraction using an 80 keV photon contact irradiator (X-Strahl). After irradiation, cells were maintained for 10 days under hypoxia 1% O_2_ and 37 °C. Colonies were revealed by crystal violet staining and counted. 

DIPG-007 cells were seeded in 1.5% soft agar containing 0, 0.05, or 0.1 µM Evo into 12-well culture plates (500 to 4000 cells/well) and placed under hypoxia (1% O_2_). After 24 h of incubation, cells were irradiated with 0, 1, 2, 3, or 4 Grays as a single fraction using an 80 keV photon contact irradiator (X-Strahl). After irradiation, cells were maintained for 10days under hypoxia 1% O_2_ and 37°C. Colonies were revealed by MTT (Thiazolyl Blue Tetrazolium Blue) staining and counted.

The plating efficiency (PE) is the ratio (number of colonies at 0 Gray/number of seeded cells), and the survival fraction (SF) is the ratio (number of colonies after irradiation/(PE × number of seeded cells)). The results were represented as a survival curve (SF depending on the irradiation dose) on a logarithmic scale. 

### 2.7. Statistical Analysis

IC_50_ values are presented as the mean ± Standard Deviation. The statistical significance was evaluated with Student’s *t*-test. Irradiation survival rates were compared with Student’s *t*-test. A *p* < 0.05 was considered as significant.

## 3. Results

### 3.1. Evo Inhibits the Growth of Pediatric Glioma Cell Lines under Hypoxia 

**Figure 1:**
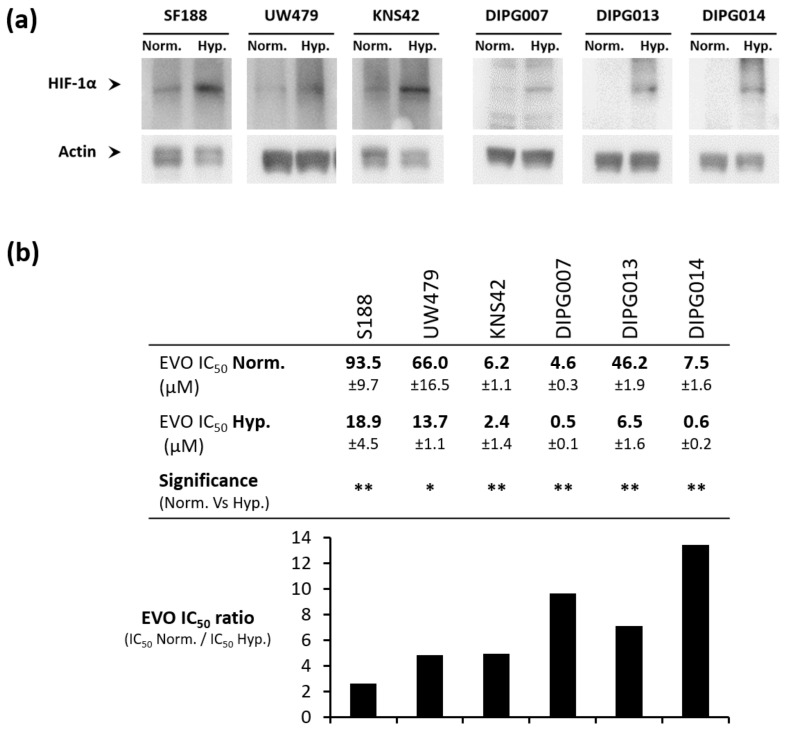
Hypoxic pediatric high-grade glioma (pHGG) and diffuse intrinsic pontine glioma (DIPG) cells are more sensitive to Evofosfamide (Evo) compared to normoxic ones. (**a**) Hypoxia inducible factor-1α (HIF-1α) expression level assessed by Western blot in pHGG and DIPG cell lines incubated for 72 h under hypoxia 1% O2 (Hyp.) compared to normoxia (Norm.). (**b**) Determination of Evo IC_50_ (inhibitory concentration 50) value after 72 h of treatment in pHGG and DIPG cell lines. IC_50_ are compared for each cell line between the normoxic condition (Norm.) and hypoxic condition 1% O2 (Hyp.) to calculate an Evo IC_50_ ratio. Values are the means of three independent experiments +/− SD. ** *p* < 0.001; * *p* < 0.05.

Considering the mechanism of Evo reduction into its cytotoxic metabolite under hypoxic conditions, we first checked that pHGG and DIPG cells acquire a hypoxic status following 72 h of incubation under 1% O_2_. To do that, we measured by Western blot the expression level of hypoxia inducible factor-1α (HIF-1α), which is degraded under normoxia and stabilized under hypoxia, and acts as a master regulator of cellular hypoxia. In the six tested cell lines (three pHGG cell lines and three DIPG cell lines), HIF-1α expression was higher when cells were incubated under hypoxia compared to normoxic conditions, indicating that they actually sense our low-oxygen in vitro conditions (Figure 1a, uncropped version in Figure S1). Nevertheless, we did not observe any cell culture suffering up to 96 h under these hypoxic conditions, as monitored by microscopy (Figure S2).

We then investigated how Evo impacts pediatric glioma cell growth. Evo inhibited cell growth in all the six cell lines tested, with reproducible Evo IC_50_ values, but a high heterogeneity in cell sensitivity among the different cell lines (Figure 1b). Most importantly, in all tested cell lines, Evo IC_50_ values were significantly decreased under 1% O_2_ hypoxia compared to normoxic conditions (Figure 1b), thus validating its action as a hypoxia-activated prodrug. Thus, for pHGG cell lines, Evo cytotoxicity appeared 2.6, 4.8, and 5 times higher under hypoxia compared to normoxia in SF188, UW479, and KNS42 cells, respectively. In DIPG cell lines, this gain in cytotoxicity was higher (9.6, 7.1, and 13.5 in DIPG-007, DIPG-013, and DIPG-014, respectively), indicating an even better benefit under hypoxia.

### 3.2. Evo Induces Cell Death after 48 h of Treatment under Hypoxia

**Figure 2:**
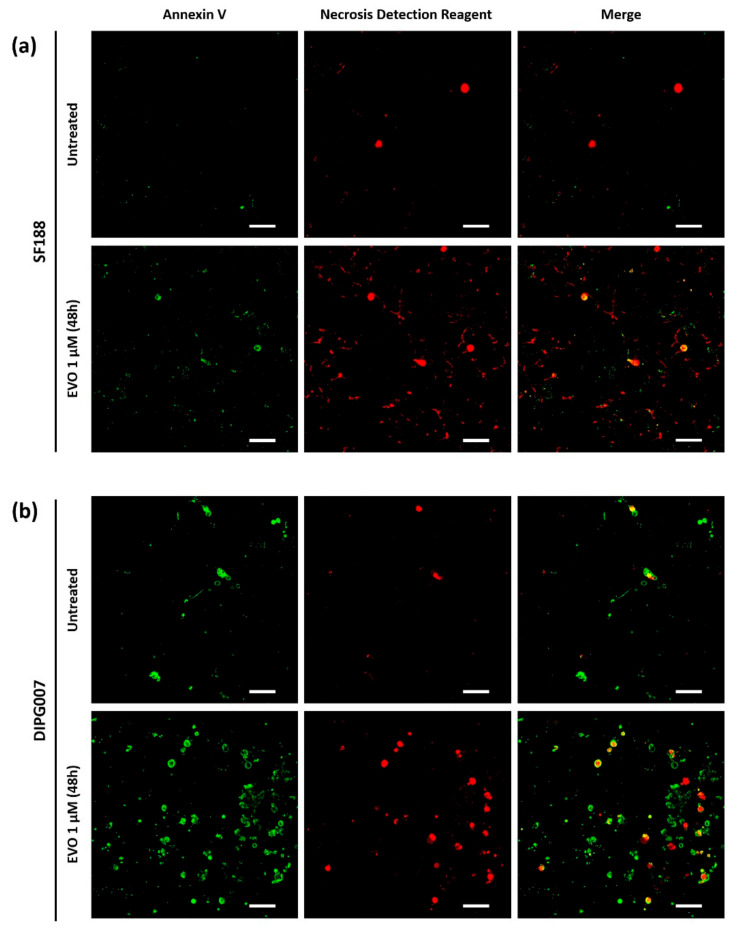

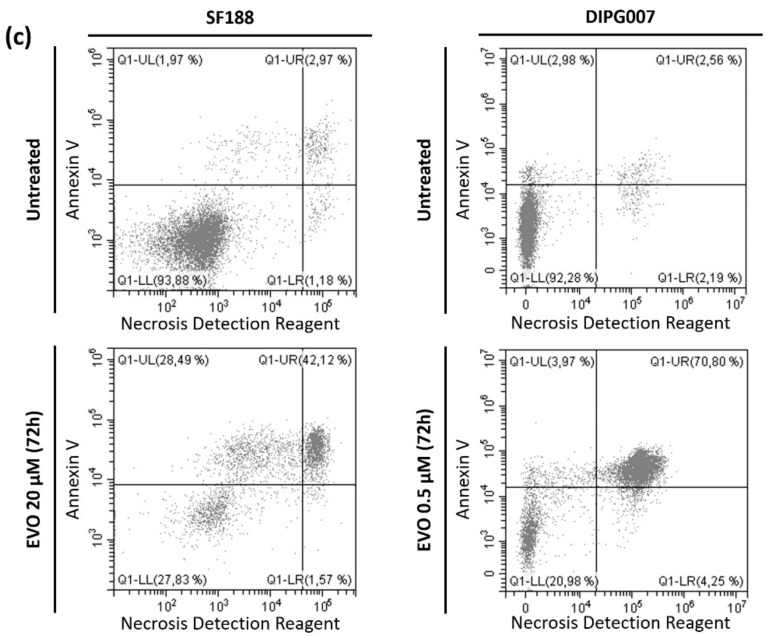
Apoptosis/necrosis detection in (**a**) SF188 and (**b**) DIPG007 cells after treatment with Evo. After treatment with 20 µM Evo for 48 h under hypoxia 1% O_2_, apoptotic and necrotic cells were stained with a cell death fluorescent detection kit and observed by confocal microscopy. Scale bar 50 µm. (**c**) Quantification performed by flow cytometry after 72 h treatment under hypoxia using IC50 Evo concentration. Results are representative of three independent experiments.

We then wondered whether Evo triggered cell death or not under hypoxia. We chose to focus on the most widely used cell line of each category, namely SF188 for pHGG and DIPG007 for DIPG, both treated with the unique dose of 1 µM Evo, which is close to the DIPG007 Evo IC50. Apoptosis/necrosis staining analyzed by microscopy revealed that, after 48 h of treatment with 1 µM Evo under hypoxia 1% O_2_, most of the SF188 pHGG cells were positive for both stains, which is indicative of a late-stage apoptosis or of necrosis (Figure 2a). As a control, untreated cells mostly did not show any significant staining. The same kind of results was observed in DIPG007 treated cells under hypoxia, with the additional presence of a subpopulation of annexin V-positive only cells (Figure 2b). Thus, in both tested cell lines, Evo induced early- and/or late-stage apoptosis at 48 h of treatment. Under IC50 conditions, i.e., 20 µM Evo for SF188 and 0.5 µM for DIPG007 during 72 h under 1% O_2_ hypoxia, flow cytometry analysis revealed a predominance of late-apoptotic and/or necrotic cells, with 42.1% and 70.8% of Annexin V^pos^/ NDR^pos^ cells in the SF188 and DIPG007 cells, respectively.

### 3.3. Evo Cytotoxic Effect Is Synergistic with Etoposide, Doxorubicin, and SN38

**Figure 3:**
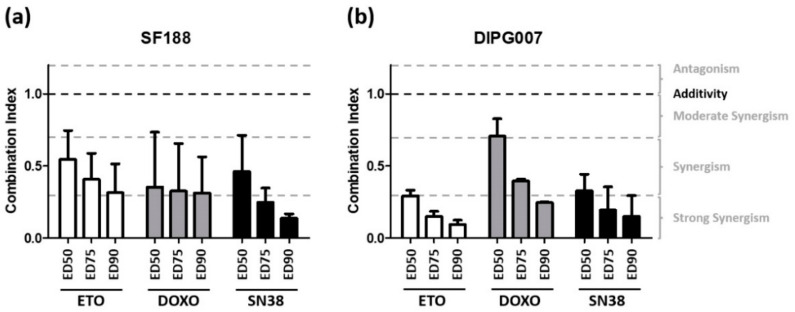
Determination of synergy between Evo and cytotoxic agents. The synergistic effect of Evo was evaluated in combination with etoposide (white), doxorubicin (grey), and SN38 (black) in SF188 (**a**) and DIPG cells (**b**). Cytotoxicity was determined after 72 h of drug exposure under 1% O_2_ and the synergistic effect was analyzed by Chou and Talalay’s method. Histograms represent the mean of three independent experiments ± SD. Results are presented as combination index (CI) at ED50, ED75, and ED90. A combination was considered as synergistic if CI < 1.

Subsequently, we tested the combination of Evo with three cytotoxic drugs widely used in pediatric oncology, frequently associated with an alkylating agent and belonging to different classes of antitumor drugs: doxorubicin (DNA intercalating agent and topoisomerase II inhibitor), etoposide (topoisomerase II inhibitor), and SN38 (topoisomerase I inhibitor). For each combination performed in both SF188 and DIPG007 cell lines, Chou and Talalay’s method revealed a significant synergistic effect, with CI mean values between 0.08 and 0.7, regardless of the dose effect (ED) considered (Figure 3). Interestingly, Evo exhibited a powerful synergy with both Etoposide and SN38 in DIPG007 cells. 

### 3.4. Evo Improves the Efficiency of Ionizing Radiations in SF188 Cell Line

Given the major role of radiation therapy in the standard treatment of pediatric gliomas, we evaluated the radiosensitizing potential of Evo in pHGG and DIPG cells. We performed a clonogenic assay on SF188 and DIPG007 cell lines exposed to low doses (0.05 µM or 0.1 µM) of Evo in hypoxic conditions, in combination with single-dose or fractionated irradiation schedule.

**Figure 4:**
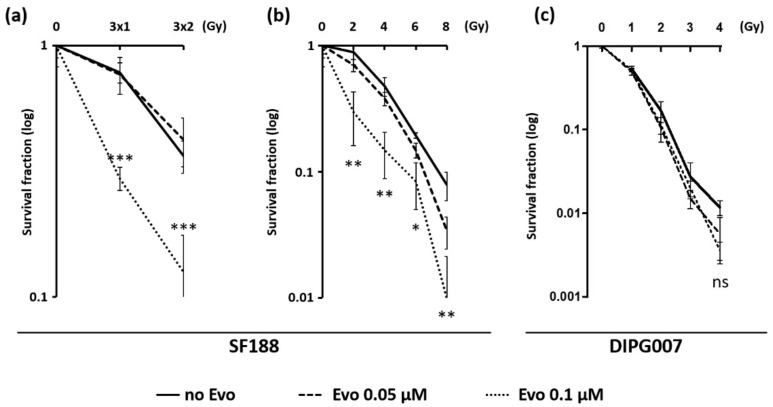
Radiosensitizing effect of Evo on SF188 and DIPG007 cell lines. The combination effect of Evo with ionizing radiation was determined by a clonogenic assay. Cells were treated with 0, 0.05, or 0.1 µM Evo under 1% O_2_ hypoxia and irradiated six hours later. (**a**) SF188 cells were irradiated with fractions of 0, 1, or 2 Grays each day for three consecutive days or (**b**) with 0, 2, 4, 6, or 8 Grays as single doses. (**c**) DIPG007 cells were irradiated with 0, 1, 2, 3, or 4 Grays as single fractions (**c**). After 10 days under 1% O_2_ hypoxia, colonies were counted, and the survival fraction was calculated for each condition. Results are means values +/− SD and are representative of three independent experiments. *** *p* < 0.0001; ** *p* < 0.001; * *p* < 0.05.

In SF188 cell line, 0.05 µM Evo did not significantly alter the survival fraction of irradiated cells. However, we demonstrated a significant difference in the survival fraction between untreated cells and cells treated with 0.1 µM Evo. We showed that 0.1 µM Evo treatment has a radiosensitizing effect in hypoxic SF188 cells exposed to a fractionated (3 × 1 Gy and 3 × 2 Gy) irradiation schedule (Figure 4a), as well as exposed to a 2–8 Gy single-dose irradiation schedule (Figure 4b). In contrast, even if Evo seemed to slightly sensitize the DIPG007 cells to ionizing radiations, no statistically significant difference was observed between 0, 0.05, and 0.1 µM Evo survival curves following a single-dose irradiation schedule (Figure 4c). Interestingly, DIPG007 cells did not support the 3 × 1 and 3 × 2 Gy fractionated irradiation schedule (no colony formed) and the survival fraction was already reduced by almost 90% with a single dose of 2 Gy, indicating a high sensitivity to ionizing radiations in vitro.

## 4. Discussion

Despite intensive and multidisciplinary management, the prognosis of children with high-grade glioma remains very poor, requiring new therapeutic approaches. Most children treated for an HGG or DIPG experience a disease relapse a few months after the initial treatment. The presence of hypoxic areas in pHGG and DIPG, as well as in many tumor types, might participate in chemo- and radio-resistance [13,22,23]. Thus, different strategies targeting these hypoxic regions have been developed for many years as the use of hypoxia-activated prodrugs (HAPs) [24]. Among them, tirapazamine was one of the first widely evaluated in clinical trials until the development of second-generation HAPs such as Evofosfamide. The use of these HAPs allows for the administration of intensified doses at the tumor site, while limiting the systemic toxicity of the molecule [11]. The results of phase II studies using Evo when combined with other drugs in different types of advanced cancers in adults (such as soft tissue sarcoma, pancreatic cancer, non-small cell lung cancer, glioblastomas, etc.) are promising [19,20,25]. Here, we demonstrated for the first time the therapeutic potential of Evo, used as a single agent or in combination, against a large panel of pHGG and DIPG cell lines. 

To validate our experimental conditions, we first checked whether cells sensed hypoxia by analyzing the HIF-1α accumulation after 48 h under 1% O_2_. This result validated the use of these hypoxic conditions for further experiments. Meng previously showed that Evo cytotoxic activity is inversely related to the oxygen rate [14]. Indeed, Evo is a second-generation HAP and its cytotoxicity is optimal at very low O_2_ concentrations, up to anoxia (for a short period). However, we chose to perform our in vitro experiments under a 1% O_2_ level, which induces a relevant cellular hypoxia, while allowing for adequate cell proliferation. Furthermore, considering the intratumoral oxygen level measured in gliomas between 0.1% and 2.5% O_2_ [10], the results obtained in our study at 1% O_2_ lead us to believe in a higher clinical relevance.

We then determined the IC_50_ values of Evofosfamide under normoxic versus hypoxic conditions in our three pHGG and three DIPG cell lines. We showed a significant variability between the different cell lines regarding Evo IC_50_ values, whether under hypoxia or normoxia. This can be partially assigned to a variability between cell lines with respect to their sensitivity to alkylating drugs. In this sense, the study of Meng and colleagues conducted on a panel of 32 cancer cell lines had also shown a significant variability of Evo cytotoxicity, under normoxia as well as under anoxia, between different types of cancer, but also between different cell lines from the same type of cancer. In that study, the Evo IC50 under hypoxia ranged from 0.1 µM in H460 lung cancer cells to 90 µM in U87MG glioblastoma cells. As expected, the Evo IC50 values under normoxia were, for all cell lines tested, much higher than in hypoxia (from 40 µM to 1.4 mM). Beyond the lineage-specific survival to alkylating agents, several other mechanisms were described to be possibly involved in Evo’s activation and effect. Among them, the reductase activity of the cytochrome P450 reductase (POR), which is necessary for Evo activation under hypoxia, can differ according to the model [14]. Recently, a study concluded that POR expression level was a major determinant for SN30000 activity, a new HAP, in three different cancer cell lines. Thus, POR expression level could be a predictive value of HAP efficiency in hypoxic tumors [26].

Despite this heterogeneity, we observed in all pHGG and DIPG cell lines tested a significant increase of Evo cytotoxicity under hypoxia compared to normoxic conditions. Indeed, Evo appeared about 3- to 5-fold more cytotoxic under hypoxia in pHGG cell lines and about 7- to 13-fold more cytotoxic under hypoxia in DIPG cell lines compared to normoxic conditions. This can be compared with the Evo IC_50_ ratio of 11 (normoxia versus anoxia) found by Meng and collaborators in the U87-MG adult glioblastoma cell line [14].

This cytotoxicity was associated with the execution of cell death processes, mainly necrosis or late apoptosis in both cell lines.

The current first adjuvant treatment of pHGG after surgical resection remains radiotherapy. Few chemotherapies have a small place in second-line treatment, mainly temozolomide (TMZ), an alkylating agent. Nevertheless, the pHGG chemosensitivity remains very low and the results of some Children’s Oncology Group (COG) studies are conflicting. In the ACNS0126 trial, the adjunction of concomitant TMZ with radiotherapy followed by adjuvant TMZ did not show a survival rate improvement [27]. However, in the recent ACNS0423 study, the addition of alkylating agent Lomustin to TMZ and radiotherapy appeared to confer a significant survival benefit [28]. Thus, Bouffet emphasized the important role of old chemotherapy drugs as alkylating agents used in combination in pHGG treatment [29]. In this frame, since Evo is converted into a Br-IMP alkylating compound, it could be envisioned as a second-line treatment in combination with other anticancer drugs or in association with radiotherapy as a radiosensitizing agent. 

With this in mind, we evaluated the synergistic potential of Evo combined with three cytotoxic drugs widely used in pediatric oncology: etoposide, a topoisomerase II inhibitor, doxorubicin, a DNA intercalating agent and topoisomerase II inhibitor, and SN38, the active metabolite of irinotecan and a topoisomerase I inhibitor. These three molecules are commonly associated with alkylating drugs. For example, the combination of irinotecan with temozolomide and bevacizumab was recently shown to prolong the survival of children with DIPG when compared to temozolomide used as a single agent [30].

Based on the statistical method of Chou and Talalay [21], we showed a significant synergistic cytotoxic effect of Evo combined with etoposide, doxorubicin, and SN38 under hypoxic conditions, against both the pHGG and DIPG cells tested. These results are consistent with previous studies, which demonstrated an antitumor effect of Evo used in combination with doxorubicin in a mouse model of non-small cancer cell lung xenograft [16]. Evofosfamide and doxorubicin have been used in combination in a clinical phase III trial in the treatment of soft tissue sarcomas in adults, and the final results of this trial are pending. Here, we highlight for the first time a synergy of Evo combined with three different chemotherapeutic drugs against pediatric gliomas.

The standard treatment of childhood HGG and DIPG is currently ionizing radiations; thus, we evaluated the radiosensitizing potential of Evo [5,31]. It is now renowned that hypoxia promotes the radioresistance of cells [13,32,33,34]. Indeed, the radiation dose needs to be 2- to 3-fold higher under hypoxia to induce the same amount of DNA damage as in normoxia [13]. Besides, Takakusagi et al. described that radiotherapy or Evo treatment, performed on subcutaneous xenografts in mice, had little impact, whereas their combination significantly increased DNA damage and necrosis in hypoxic areas [32]. Moreover, the active form of Evo is part of the alkylating agent family, described to provide a radiosensitizing effect in different models [35]. Interestingly, in a syngeneic rat model, Stokes and collaborators showed that 18-fluoromisonidozole Positron Emission Tomography (PET) imaging, sensitive to tumor hypoxia and perfusion, was able to predict the response to Evo [36].

Here, we demonstrated that Evo radiosensitizes hypoxic SF188 pHGG cells with doses as low as 2 Gy when using a single-dose schedule, and 3 × 1 Gy when using a fractionated schedule. In contrast, despite a tendency towards an Evo-induced radiosensitization in DIPG007 cells, no statistically significant effect was measured. This is probably related to the higher sensitivity of these cells to ionizing radiations in vitro, which impaired us to further study this aspect. A study by Peeters also showed an antitumor effect of Evo combined with radiotherapy in two mouse tumor models (rhabdomyosarcoma and lung cancer) [37]. This effect was inversely correlated to the level of oxygenation in the tumor. In our study, we chose to use conventional radiotherapy to establish our proof of concept. In the future, it would be interesting to test the combination of Evo with proton therapy or carbon ion beam, which represent promising alternatives to reduce the radiation exposure, and could be particularly relevant in pediatric CNS (Central Nervous System) malignancies [34].

We injected our two representative cell lines in mice to establish subcutaneous xenografts, but DIPG7 cells did not develop tumors, and SF188 tumors did not display hypoxic markers in this setting, which prevented us from assessing the antitumorigenic efficiency of Evo in vivo. Nevertheless, preclinical and clinical investigations carried out in glioblastoma make us confident concerning the potential of Evo in vivo. Indeed, Evo appeared to cross the BBB (Blood Brain Barrier), which is one of the main obstacles during the transfer from preclinical to clinical application in the brain context. Additionally, as mentioned, there is a correlation between brain tumor hypoxia and Evo efficiency, demonstrated by Stokes and collaborators. Given the direct Evofosfamide effects that we report in hypoxic pediatric glioma cells, and the fact that pediatric brain tumors present a highly hypoxic profil by MRI (Magnetic Resonance Imaging), we presume that HAPs as Evo should exhibit a significant effect on pediatric HGG and DIPG tumors.

Very recently, the formulation of Evo within microspheres proved to enhance its antitumoral efficacy against tumors implanted in the liver of rabbits [38], which opens the perspective for a better delivery of Evo to tumors.

Of note, another category of aggressive pediatric gliomas, namely Posterior fossa A ependymomas, was shown to be highly dependent on hypoxia-driven metabolic and epigenetic mechanisms [39], and the treatment of these tumors may therefore also benefit from the use of Evo.

Altogether, these results strongly support that Evo represents an interesting novel weapon within our arsenal to fight pediatric gliomas.

## 5. Conclusions

In conclusion, we report the first preclinical proof of evidence about the efficiency of a novel hypoxia-activated prodrug, Evo, in pediatric HGG and DIPG. Interestingly, Evo appears effective in hypoxic glioma cells. The synergistic effects observed with SN38, doxorubicin, and etoposide, as well as the radiosensitizing effect, are of interest for pediatric HGG and DIPG treatment.

## Data Availability

The data presented in this study are available on request from the corresponding author.

## Supplementary Materials

The following are available online at https://www.mdpi.com/article/10.3390/cancers13081804/s1, Figure S1: Uncropped Western blot against HIF-1α on pHGG and DIPG cell lines incubated for 72 h under hypoxia 1% O2 (Hyp.) compared to normoxia (Norm.), Figure S2: Visual aspect and viability of cell culture after 96 h incubation under 1% O_2_ hypoxia compared to normoxia, Figure S3: IC_50_ of ETO, DOXO, and SN38 after 72 h of treatment in SF188 and DIPG007 cell lines under hypoxic condition 1% O_2_.
