# Supplementary material for: Evofosfamide Is Effective against Pediatric Aggressive Glioma Cell Lines in Hypoxic Conditions and Potentiates the Effect of Cytotoxic Chemotherapy and Ionizing Radiations
Source: Cancers (Basel). 2021 Apr 9;13(8):1804. doi: 10.3390/cancers13081804 (PMC8070185; doi:10.3390/cancers13081804)
Supplement: Supplementary file 1 [file cancers-13-01804-s001.pdf]

cancers

MDPI

Supplementary Materials

Evofosfamide Is Effective against Pediatric Aggressive Glioma Cell Lines in Hypoxic Conditions and Potentiates the Effect of Cytotoxic Chemotherapy and Ionizing Radiations

Quentin Bailleul, Pauline Navarin, Mélanie Arcicasa, Christine Bal-Mahieu, Angel Montero Carcaboso, Xuefen Le Bourhis, Alessandro Furlan, Samuel Meignan and Pierre Leblond

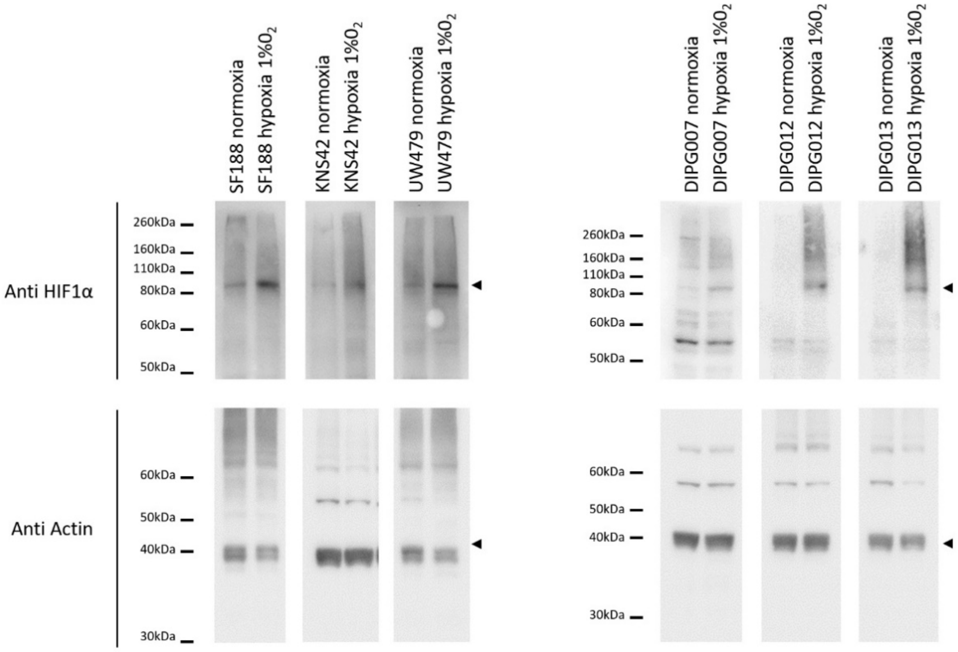

Figure S1. Uncropped western blot against HIF-1*α* on pHGG and DIPG cell lines incubated for 72 h under hypoxia 1% O_2 (Hyp.) compared to normoxia (Norm.). Actin was used as loading control.

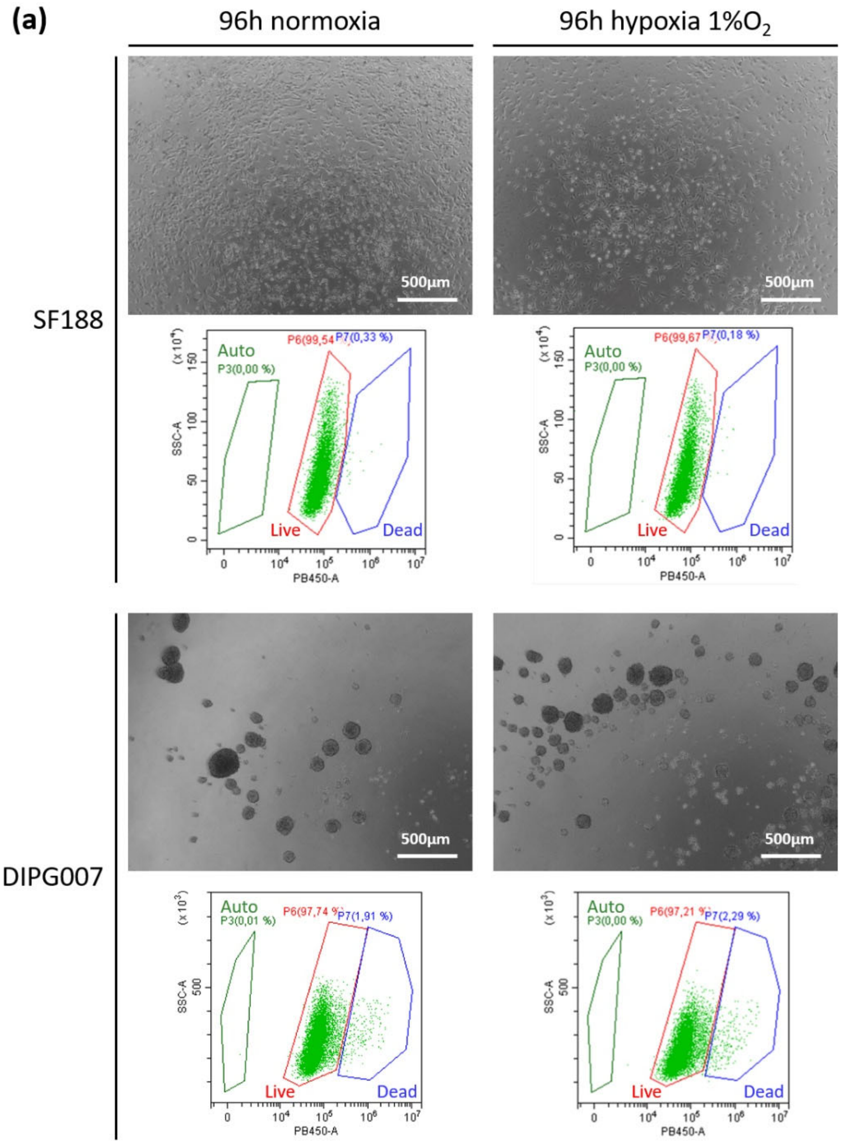
(a)
96h normoxia
96h hypoxia 1%O2
SF188
500µm
500µm
Auto
P3(0,00 %)
P6(99,54
P7(0,33 %)
P6(99,67
P7(0,18 %)
Live
Dead
SSC-A
PB450-A
DIPG007
P3(0,01 %)
P6(97,74 %)
P7(1,91 %)
P6(97,21 %)
P7(2,29 %)

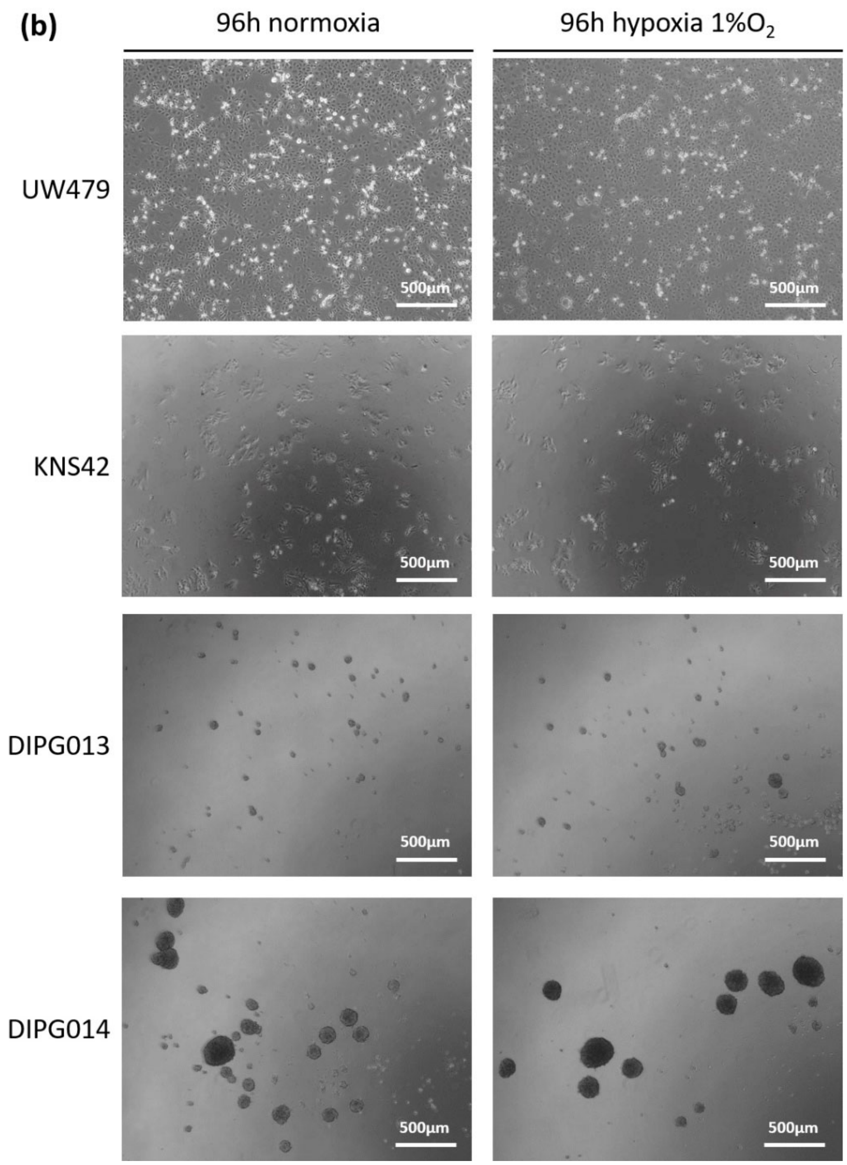

Figure S2. Visual aspect and viability of cell culture after 96h incubation under 1%O_2 hypoxia compared to normoxia. Cells were grown under hypoxia or normoxia for 96h. Then, brightfield microscope pictures of the global cell populations were taken (**a** and **b**). At the same time, the viability of SF188 and DIPG007 was evaluated by flow cytometry using Live/Dead staining (**a**). The green gate corresponds to cell autofluorescent without labeling. Red gate delimits live cells, and bleu gate delimits dead cells. Percentages represent the rate of cells from the total population in each gate. Results are representative of triplicates.

	SF188	DIPG007
ETO IC_{50} Hyp. (µM)	**6.2** ±0.6	**21.9** ±5.7
DOXO IC_{50} Hyp. (µM)	**0.33** ±0.01	**0.1** ±0.01
SN38 IC_{50} Hyp. (nM)	**107,1** ±4.6	**6.9** ±1.5

Figure S3. IC_{50} of ETO, DOXO, and SN38 after 72h of treatment in SF188 and DIPG007 cell lines under hypoxic condition 1% O2. Values are the means of three independent experiments +/−SD.
